# Treatment of Piles

**Published:** 1883-04-20

**Authors:** J. G. Westmoreland

**Affiliations:** Atlanta, Ga.


### TREATMENT OF PILES.

BY J. G. WESTMORELAND, M. D., ATLANTA, GA.

Considering the promptness and certainty of curing piles, by injection, it is remarkable that such a large number of cases should now exist. I once attempted to cure by ointments, ligature or the ecraseur. With these painful, tedious and uncertain modes of treatment, it is not strange that those thus afflicted should hesitate to be treated. I have now abandoned all other means and adopted the plan of injection which I advocated during my editorial connection with the Atlanta Medical and Surgical Journal.

I succeed in permanent cure by this method in less than a week, without pain, to any extent, or confinement to bed, necessarily. Why physicians generally do not adopt this plan and relieve the immense amount of suffering and prevent or cure the results of impotence and melancholy which so frequently follow, is hard to determine.

No claim is made to originality in this plan of treatment, but experience in its use has led to more speedy and certain cure than was had years ago. Carbolic acid, the great remedy for this disease, was at first used in a diluted form by being mixed with olive oil, and, even then, caution was advised by practitioners, lest serious results should follow. It is now known that the undiluted acid may be injected into the hemorrhoidal tumors, not only with safety, but with the effect of speedy and permanent cure.

During last year I was called upon to prescribe for a gentleman suffering very much from inflamed hemorrhoids. I hesitated to adopt the curative plan with such inflammation of the parts, but finding no relief from soothing applications for a day or two, with some misgivings, the injection of pure carbolic acid, at five or six points was made, with the effect of immediately lessening the suffering and of permanent cure of the piles in a few days.

Compared with the ligature and excision by the ecraseure, injection is perhaps not more hazardous, and infinitely less painful, and equally, if not more certain of cure.

The abominable practice of ligating is not only barbarously painful, but more tedious and dangerous than any other mode of treatment usually adopted. Non-medical men starting out as pile curers succeed in getting subjects willing to be tortured for ten or fifteen days by a cord around the tumors, and then pay largely for the fun. (?) This is because the people know nothing of a better plan.

